# Environmental stressors and sleep health among low-income older adults: a mixed-methods study

**DOI:** 10.1093/geroni/igaf122.4321

**Published:** 2025-12-31

**Authors:** Shuxian Hua, Youngmin Cho, Sofia Liu, Hannah Fu, Michelle Liu, Claire Wang, Russell Calderon, Junxin Li

**Affiliations:** University of North Carolina at Chapel Hill, Chapel Hill, North Carolina, United States; Johns Hopkins University, Baltimore, Maryland, United States; Johns Hopkins University, Baltimore, Maryland, United States; Johns Hopkins University, Baltimore, Maryland, United States; Johns Hopkins University, Baltimore, Maryland, United States; Johns Hopkins University, Baltimore, Maryland, United States; Johns Hopkins School of Medicine, Baltimore, Maryland, United States; Johns Hopkins University, Baltimore, Maryland, United States

## Abstract

Environmental factors are increasingly recognized as critical determinants of sleep health, particularly among older adults in low-income communities. This convergent mixed-methods study aimed to: (1) explore sleep environment stressors (SES) reported by community-dwelling, low-income older adults; and (2) compare subjective sleep health between those affected by SES and those who were not. Twenty-four older adults participated. SES were measured with Assessment of Sleep Environment (ASE), a 4-point Likert scale from “Strongly Agree” to “Strongly Disagree”. Participants were classified as Environmentally Impacted Sleepers (EIS, n = 14) if they endorsed any AES items, or as Environmentally Unimpacted Sleepers (EUS, n = 10) if they did not. Sleep health was assessed with the Pittsburgh Sleep Quality Index (PSQI), with group comparisons analyzed using Welch’s t-tests and effect sizes (Cohen’s d with Hedges’ correction). Semi-structured interviews further explored perceived environmental stressors and sleep experiences, which were inductively coded and thematically analyzed. The most frequently self-reported stressors were: “too soft mattress” (n = 7), “uncomfortable smell” (n = 6), “too noisy” (n = 5), “too humid” (n = 5), “too cool” (n = 5), “too much light” (n = 5). Qualitative findings revealed that outdoor noise and thermal conditions as major stressors, and participants noted that adjusting their environment (e.g., lighting, sound, or fans) improved sleep; most participants described a cool, dimly lit room with some background noise as ideal. EIS group had worse sleep health (p<.001, d = 1.87). Both groups perceived poor sleep quality, frequent night awakenings, and medical aid use; yet EIS group more commonly described challenges returning to sleep. Addressing environmental stressors is essential for healthy aging sleep.

